# Evaluating Welfare, Milk Quality and Yield of Rendena Cows in Loose vs. Tied Housing Systems

**DOI:** 10.3390/ani16040636

**Published:** 2026-02-17

**Authors:** Silvia Sabbadin, Silvia Magro, Angela Costa, Valentina Lorenzi, Francesca Fusi, Luigi Bertocchi, Massimo De Marchi

**Affiliations:** 1 Department of Agronomy, Food, Natural Resources, Animals and Environment, University of Padova, 35020 Legnaro, Padova, Italy; silvia.sabbadin.4@phd.unipd.it (S.S.); massimo.demarchi@unipd.it (M.D.M.); 2 Department of Veterinary Medical Sciences, Alma Mater Studiorum University of Bologna, 40064 Ozzano dell’Emilia, Bologna, Italy; angela.costa2@unibo.it; 3 Italian Reference Centre for Animal Welfare, Istituto Zooprofilattico Sperimentale della Lombardia e dell’Emilia Romagna “Bruno Ubertini”, 25124 Brescia, Italy; valentina.lorenzi@izsler.it (V.L.); francesca.fusi@izsler.it (F.F.); luigi.bertocchi@izsler.it (L.B.)

**Keywords:** ClassyFarm, local breed, alpine breed, dual-purpose cows, free stall, tie stall, management, individual milk, protein content

## Abstract

The objectives of this study were (i) to analyze the ClassyFarm welfare scores in loose housing system (LHS) and tied housing system (THS) Rendena herds, and (ii) to investigate the influence of the housing on individual milk yield and quality traits in Rendena cows. The dataset consisted of 3761 individual milk samples from 750 Rendena cows, collected between August 2022 and November 2023 from 17 single-breed herds of the Veneto region. Available data included days in milk (DIM), parity, and milk yield, as well as fat, protein, casein, and lactose contents, somatic cell count, differential somatic cell count, and urea concentration. For milk traits, a linear mixed model included housing system, DIM, and parity as fixed effects, while cow, herd-test-date, and residual variability were random effects. In both housing systems, a good level of welfare was observed, although shortcomings in biosecurity measures were identified in both LHS and THS. Milk quality (in terms of protein % and casein %) and yield were higher in LHS compared to THS. The observed differences cannot be explained by the housing system alone, as other management and nutritional factors may have played a role, highlighting the need for further studies to clarify these contributions.

## Simple Summary

Local cattle breeds often rely on small farms with limited resources, making it essential to ensure good animal welfare and high-quality food production. In northeastern Italy, the Rendena cattle breed is commonly raised in either loose housing, which allows cows to move freely, or under tied housing, typical of mountain areas, in which cows are tethered in winter and have pasture access. This study aimed to evaluate animal welfare in these two housing systems and to assess whether the type of housing affects milk production and milk quality. Data were collected from 750 Rendena cows on 17 farms over a 15-month period. Overall, both housing systems showed good levels of animal welfare; however, cows housed in loose systems produced more milk with higher protein and casein contents compared to cows kept in tied systems, and these parameters are crucial for cheese making. Further research is needed to understand whether housing systems and other factors, such as nutrition or management, can help Rendena farmers improve milk production, supporting their work and the conservation of the breed.

## 1. Introduction

Alpine local cattle breeds, such as the Rendena, represent a strategic resource for sustainable livestock systems [1]. Besides their agricultural role and ecosystem service, these local breeds contribute significantly to the resilience of alpine communities by supporting economic opportunities and preserving cultural heritage and traditions [1].

The Rendena is a dual-purpose cattle breed native to the mountainous regions of north-eastern Italy [2]. According to the Italian Breeders Association (AIA), the Rendena population in 2024 consisted of 3428 cows distributed across 174 herds monitored for milk production and quality, with Veneto (47%) and Trentino Alto Adige (21%) hosting most of the animals [3]. The national association of Rendena breeders, Associazione Nazionale Allevatori Bovini di Razza Rendena (ANARE, http://www.anare.it, accessed on 31 July 2025), based in Trento, is in charge of the herd book of the Italian population. The Rendena breed is characterized by a medium to small body size, strong adaptability to grazing conditions, and valuable functional traits, i.e., fertility and longevity [2]. Despite its moderate milk yield, averaging around 5000 kg per lactation, the breed exhibits favorable reproductive performance and maintains a good beef conformation [1].

A minority of the Rendena cattle population is still raised under traditional, low-input systems, which are typically small-scale and located in mountain areas [4,5]. These traditional systems are usually organized in tied housing systems (THS) due to geographical and spatial constraints [6]. Within this framework, THS have persisted in traditional farming practices in the Italian alpine areas, often integrated into semi-extensive farming models in which cows spend most of the year grazing on pastures and are tethered indoors only for limited periods [7]. This seasonal balance enables animals to benefit from outdoor access while maintaining traditional husbandry methods [8]. However, the use of THS remains a topic of debate at both national and European levels, as, in contrast with the 5 freedoms for welfare, they restrict the natural movements and behavior of cows and increase the risk of several health issues [7,9]. Ensuring that cows can move freely and comfortably is essential for their welfare [10].

Today, animal welfare is a central focus in dairy farming as it contributes to disease prevention, reduces antimicrobial use, and enhances both milk quality and production [11]. In Italy, the General Directorate of Animal Health and Veterinary Drugs (DGSA) of the Ministry of Health assigned the development of a standardized national system for evaluating animal welfare to the Istituto Zooprofilattico Sperimentale della Lombardia e dell’Emilia Romagna (IZSLER), which hosts the National Reference Centre for Animal Welfare (CReNBA) [12]. This national system, called ClassyFarm, is used by trained veterinarians to conduct farm inspections and, subsequently, to calculate a welfare score based on comprehensive evaluations of multiple farm aspects, i.e., biosecurity, management, structures, and animal-based measures [11]. The ClassyFarm system can be applied to several animal species and categories, including dairy and beef cattle. Authorized veterinarians assess dairy farms using standardized checklists specifically designed for either loose housing systems (LHS) or THS. The collected data are then entered into the ClassyFarm web-based platform (https://www.classyfarm.it, accessed on 30 September 2024), where a dedicated algorithm processes the collected information to generate an overall welfare score ranging from 0% (poor welfare) to 100% (excellent welfare) [13].

To date, information on welfare in Rendena cattle under LHS and THS is poorly documented, whereas similar investigations have already been conducted in more widely distributed breeds such as Holstein and Simmental cattle. Evaluating the level of welfare of Rendena herds is essential for improving farm profitability and consumer perception, supporting breed conservation, and enhancing product quality (mainly milk and meat). Based on the findings reported in other cattle breeds, we hypothesized that welfare scores assessed by ClassyFarm differ between LHS and THS in Rendena herds, and that the housing system (HS) affects the individual milk yield and milk quality traits in Rendena cows. Accordingly, the objectives of our study were: (i) to analyze the ClassyFarm welfare scores achieved by LHS and THS, and (ii) to examine whether the HS influences individual milk yield and quality in Rendena cows.

## 2. Materials and Methods

### 2.1. ClassyFarm Welfare Data

The ClassyFarm scores were collected from 17 single-breed herds in the Veneto region, north-eastern Italy, with 5 herds under LHS and 12 herds under THS. One ClassyFarm assessment per farm was considered for the period between August 2022 and November 2023, as this evaluation is typically conducted once a year by trained veterinarians. During the farm inspections, veterinarians obtained information through farmer interviews and direct observations to fill the ClassyFarm checklist.

The LHS and THS checklists are specifically tailored to accommodate housing-specific conditions and evaluate all animal categories (i.e., calves, heifers, lactating cows, and dry cows) [8,14]. The LHS checklist consisted of 105 questions (hereinafter referred to as “indicators”), whereas the THS checklist included 99 indicators (Supplementary Tables S1 and S2) [12]. The indicators are divided into 5 thematic areas in both checklists: (i) biosecurity, (ii) management, (iii) structures, (iv) animal-based measures (ABMs), and (v) major hazards. Specifically, the management area examines the full range of daily activities carried out by farm operators, from proper animal handling in the barn to the milking routine (Supplementary Tables S1 and S2). The structures area evaluates the suitability and safety of housing facilities, including freedom of movement and social interaction, the availability of specific functional areas (e.g., calving and hospital pens), and microclimatic conditions (e.g., temperature and humidity). The ABMs area refers to welfare indicators that can be measured directly on the animals (e.g., body condition score, integument alterations, and mutilations) or indirectly from farm databases (e.g., annual mortality or somatic cell count (SCC)). The biosecurity area concerns the prevention of the introduction and spread of diseases through health monitoring activities or controlled access of visitors and vehicles (Supplementary Tables S1 and S2). Major hazard indicators pertain to the farm’s capacity to manage emergencies, including water or electricity outages and fires (Supplementary Tables S1 and S2).

Each indicator included in the ClassyFarm checklists is accompanied by specific guidelines for the trained veterinarian responsible for the on-farm evaluation. Indicators are assessed using either a two-level scale (poor/fair) or a three-level scale (poor/fair/good). The content and number of the indicators differ between the LHS and THS checklists, as do the weights and scores assigned to each indicator. These differences arise from an expert opinion elicitation process based on a modified Delphi technique, aimed at identifying the most relevant welfare aspects for each housing system [14].

The LHS and THS checklists are therefore structured and scored differently to account for the peculiar characteristics, management practices, and structural constraints of the two most widespread HSs in Italy. Despite these differences, both checklists adopt common scoring formulas and express results on a standardized scale ranging from 0 to 100%, where 0% indicates poor welfare and 100% indicates optimal welfare status [8,14].

The data from the completed checklist are processed and validated by the ClassyFarm IT platform [7]. The ClassyFarm algorithm calculates scores for each thematic area by summing the scores assigned to the individual indicators belonging to that area and expressing the result as a percentage on a 0–100% scale. In addition, the platform computes the overall welfare score (%) based on an arithmetic mean that integrates the score of the ABMs with the total score of the management and structures areas. In the calculation of the overall welfare score, the major hazards area is excluded, while greater weight is assigned to the ABMs, as they directly reflect animal outcomes and capture the combined effects of multiple management, environmental, and structural factors. The biosecurity score is automatically incorporated into the management area, as it is included as the final indicator within this area (Supplementary Tables S1 and S2). The algorithm and weighting procedures underlying the ClassyFarm scoring system are not publicly accessible [15].

The distributions of ClassyFarm scores were visually inspected before statistical analysis.

### 2.2. Individual Milk Data

The test-day records were retrieved from the official routine milk recording database of the Breeders’ Association of the Veneto Region (ARAV, Vicenza, Italy). To ensure temporal alignment with the welfare evaluation, only records from cows in the 17 selected herds, collected within 200 days before or after the ClassyFarm assessment date, were included.

The test-day records comprised information on days in milk (DIM), parity, and milk yield (kg/d), as well as fat, protein, casein, and lactose content (%), SCC (cells/mL), differential somatic cell count (DSCC, %), and urea concentration (mg/dL). Milk samples after collection were always preserved with Bronopol, transported to the ARAV milk laboratory, and analyzed through the CombiFoss^TM^ 7 (FOSS Electric A/S, Hillerød, Denmark). The somatic cell score (SCS) was calculated as SCS = 3 + log_2_ (SCC/100,000) to normalize the distribution of data points, and the concentration of polymorphonuclear neutrophils and lymphocytes (DSCC_N_, cells/mL) was determined from SCC and DSCC according to the formula DSCC_N_ = (SCC × DSCC)/100 [16]. The DSCC_N_ was then log_2_-transformed using the same formula applied for SCS to derive the differential somatic cell score (DSCS).

Values of milk traits deviating more than 3 standard deviations from the respective mean were treated as missing. Only cows between 5 and 400 DIM with a minimum of 3 test-day records were included in the statistical analysis to ensure repeated measurements. Additionally, only herd-test dates with at least 3 sampled cows were taken into account. The dataset used for statistical analysis consisted of 3761 test-day records from 750 lactating cows: 1944 records from 400 lactating cows in LHS and 1817 records from 350 lactating cows in THS. The distributions of milk traits were visually inspected before statistical analysis and found to be normal.

### 2.3. Statistical Analysis

Statistical analyses were conducted using the R software v. 4.4.1 [17]. Descriptive statistics of the six scores (overall welfare, biosecurity, management, structures, ABMs, and major hazards) were calculated within each HS. The analysis of variance for milk traits was performed with the R package “lme4” [18] using the following mixed linear model:

y_ijklm_ = µ + HS_i_ + P_j_ + D_k_ + C_l_ + H_m_ + e_ijklm_, (1)

where y_ijklm_ is the dependent variable (milk yield, fat content, protein content, casein content, lactose content, SCS, DSCS, urea concentration); µ is the overall intercept of the model; HS_i_ is the fixed effect of the ith HS type (i = LHS or THS); P_j_ is the fixed effect of the jth parity (j = 1, 2, ≥3); D_k_ is the fixed effect of the kth DIM (k = 8 classes, with the first being a class from 5 to 50 DIM, followed by 7 classes of 50 DIM each); C_l_ is the random effect of the lth cow (750 levels) assumed to be distributed as ~N (0, σ^2^_C_), where σ^2^_C_ is the cow variance; H_m_ is the random effect of the mth herd-test-date (112 levels) assumed to be distributed as ∼N (0, σ^2^_H_), where σ^2^_H_ is the herd-test-date variance; and e_ijklm_ is the random residual assumed to be distributed as ~N (0, σ^2^_e_), where σ^2^_e_ is the residual variance. Multiple comparisons of least square means were performed with the R packages “emmeans” [19] and “multcomp” [20], using the Bonferroni adjustment with significance set at *p* ≤ 0.05.

## 3. Results and Discussion

### 3.1. Welfare Level

The Rendena breed, recognized as Slow Food Presidium [21,22], is considered vulnerable to extinction, and its preservation is vital for promoting long-term sustainability and safeguarding genetic diversity [23]. At the same time, efforts to maintain both the productivity and welfare of Rendena cows are essential. Ensuring and demonstrating high welfare levels in local breeds like the Rendena can therefore valorize and enhance the marketability of their products [24], supporting the viability of small-scale farms in marginal mountain areas and openness to innovations and improvements [25,26]. In this context, the ClassyFarm system represents a valuable tool, as it provides a comprehensive welfare assessment for dairy herds by integrating management practices, structural conditions, ABMs, and biosecurity measures into a standardized scoring framework.

Data on herd consistency were collected during the ClassyFarm inspections for all animal groups, including lactating cows, dry cows, heifers, and calves (Table 1). The LHS herds were three times larger than the THS herds, with an average of 101 and 30 lactating cows, respectively (Table 1).

The average overall welfare score was 79.14% for LHS and 80.60% for THS, indicating a good level of welfare in both HSs (Table 2). Notably, THS farms with Rendena cattle obtained higher overall welfare scores than those reported in previous studies.

For example, Sabbadin et al. (2025) found an average overall welfare score of 72.56% in THS herds mainly composed of Holstein and Simmental cows, while Moriconi et al. (2024) reported an average overall welfare score of 76.25% in local Valdostana THS herds [7,15]. Superior welfare outcomes of Rendena herds in THS could be consistent with breed-specific characteristics (i.e., rusticity, robustness, and adaptability) and management practices [4,5]. Moreover, in the individual areas of management, structures, ABMs, and biosecurity, Rendena herds in THS outperformed the herds assessed by Sabbadin et al. (2025) [15]. A similar trend was observed when comparing the management, structures, and ABMs scores of Rendena THS herds with the THS herds described by Mauricio et al. (2025), which included both herds of specialized breeds (i.e., Holstein and Brown Swiss) and herds of local breeds (i.e., Reggiana and Modenese) [13]. Rendena cows have been raised under traditional low-input systems in mountainous regions for decades, suggesting that they are well adapted to such environments, including the debated THS. Different cattle breeds may exhibit varying levels of adaptation to specific HSs, and the differing prevalence of certain welfare issues could be influenced by breed-specific factors [27]. In many specialized dairy cow breeds, selection goals have altered the physical characteristics [27]. This, in turn, has differentiated their welfare requirements, potentially making them less suited to restrictive HSs, compared to local breeds such as Rendena. Additionally, our findings likely reflect the optimal level of commitment and management efforts of the farmers in this specific context.

Regarding the major hazards area, the average score was comparable between Rendena herds in THS and THS herds of Sabbadin et al. (2025) [15]. However, the low score observed in this study (52.72%) suggests that THS farms have room to improve their preparedness for emergencies [8].

Rendena herds in LHS recorded lower scores in biosecurity, management, structures, and major hazards than those reported by Sabbadin et al. (2025), except for ABMs, where they performed better (83.48% and 81.10%, respectively) [15]. Compared with the LHS herds described by Mauricio et al. (2025), Rendena herds achieved higher scores in structures and ABMs, while in management, they obtained lower results only when compared with specialized LHS herds fed total mixed rations [13]. These findings likely reflect the Rendena breed’s ability to adapt to LHS conditions despite less favorable structures and management practices.

In both HSs, the biosecurity was the lowest-scoring area (49.94% in LHS and 51.15% in THS; Table 2), and Rendena LHS herds showed a large score gap (~ 2.6 points) with the LHS herds of Sabbadin et al. (2025) [15]. In the alpine region of South Tyrol, Zanon et al. (2024) explored the presence of biosecurity measures and their relationship with milk quality traits in small-scale dairy farms in both HSs [28]. Suboptimal performance in the biosecurity area may reflect the difficulty of applying certain measures in small farms, where economic, logistic, and topographical limitations hinder their feasibility [28].

### 3.2. Milk Quality and Yield

The investigation of welfare, milk quality, and production of Rendena herds is crucial to confirm that traditional practices do not compromise animal health or performance. Milk from local breeds often possesses distinctive qualities, such as higher fat and protein content and superior cheesemaking properties compared to specialized breeds like Holstein [5,24]. These attributes contribute to the added value of niche dairy products, especially those associated with quality labels like Protected Designation of Origin (e.g., Spressa delle Giudicarie cheese from Rendena milk) or Protected Geographical Indication. In this context, the quality of Rendena milk is fundamental, as it supports the production of cheeses and traditional dairy products.

The descriptive statistics of individual milk traits were reported in Table 3 and were consistent with previous literature [4,29,30].

**Figure 1:**
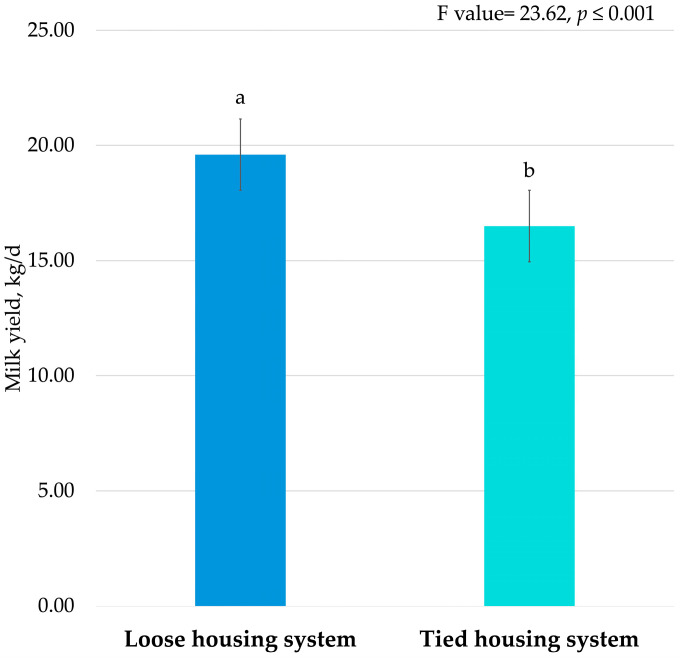
Least square means of milk yield (kg/d) for the fixed effect of housing system. Means with different superscript letters are significantly different (*p* ≤ 0.001).

The fixed effect of HS included in Equation (1) was significant for milk yield (*p* < 0.001; Figure 1), protein and casein contents (*p* < 0.01; Table 4), and urea concentration (*p* < 0.05; Table 4). DIM and parity were highly significant for all milk traits (*p* < 0.001). The Rendena cows exhibited higher milk production in LHS (19.60 kg/d; Figure 1) compared to THS cows (16.50 kg/d).

It has been reported that a total mixed ration results in higher milk yield compared to pasture-based feeding [31,32], which is likely more common in THS farms. Protein and casein contents were higher in milk from LHS cows (3.50% and 2.77%, respectively) than in milk from THS cows (3.41% and 2.70%, respectively; Table 4).

These results align with the findings of Summer et al. (2014) [33] and partially with those of Sabbioni et al. (2012), who investigated the bulk milk composition of the autochthonous Bianca Val Padana and commercial Holstein cows, fed a total mixed ration in LHS and traditional feeding in THS [34]. Bianca Val Padana cows produced less milk but with higher protein % in THS compared to LHS, whereas Holstein cows had higher milk yield in LHS, with no differences in protein % between HSs [34]. Nogalski and Momot (2023) reported no significant differences in milk protein content between HSs [35], highlighting the variability among studies and suggesting that additional factors, such as feeding, pasture access, and breed, may influence individual milk composition. For example, LHS farms are more likely to adopt total mixed rations, which promote more efficient nutrient utilization, i.e., protein content, supporting the optimal expression of dairy cattle’s genetic potential for milk quality [33].

The SCC is a well-established indicator of inflammatory processes in individual cows, of the udder health status of a herd, and the quality of raw milk [35]. Interestingly, no differences between HSs were detected concerning SCS and DSCS. The literature reports mixed findings on this topic: for example, Holstein cows milked directly in THS showed lower SCC compared to Holstein cows housed in LHS and milked in milking parlors [35]. On the contrary, Summer et al. (2014) and Sabbioni et al. (2012) reported that SCC was lower in LHS farms [33,34]. In the present study, no information was available on milking methods, milking frequency, or parlor management, which limits the interpretation of potential mechanisms underlying the observed results. Nevertheless, the favorable udder health status observed may reflect the attentive care provided by farmers, which may have played a positive role in the udder health and management of Rendena herds. Moreover, Rendena cows, like other local breeds, may benefit from inherent genetic characteristics that contribute to udder health and disease resistance [28]. In fact, the average SCS value (3.14) was similar to that previously reported for other local breeds, such as Burlina (SCS 3.06) and Alpine Grey (3.13) [36].

The LHS milk exhibited a lower concentration of urea compared to THS milk (21.70 mg/dL and 24.30 mg/dL, respectively; Table 4), whereas Nogalski and Momot (2023) reported a higher urea concentration in LHS milk [35]. Milk urea is a useful indicator of diet composition, as it reflects the balance between crude protein and energy in the diet [37]. The recommended range for milk urea concentration is 15.0–30.0 mg/dL [37], and the least square means observed in both HSs fell within this interval, suggesting properly balanced diets (Table 4). While the primary factor influencing milk urea is dietary protein content, values can also vary due to non-nutritional factors such as season, month, parity group, stage and number of lactations, and the health of the rumen and liver [37,38]. Since detailed information on diet and pasture access (yes/no) was unavailable, considerations related to milk yield and quality traits remain limited.

### 3.3. Limitations and Perspectives

As pointed out by Zanon et al. (2024) [28], the good welfare scores and cows’ performance in the two HSs suggest that, despite their inherent differences, both systems can be equally effective for the Rendena breed if properly managed. Moriconi et al. (2024) evaluated the welfare level of cows in THS belonging to the local Valdostana breed, reared in semi-extensive alpine farming systems, similar to Rendena cows [7]. They found that the farms achieved good ClassyFarm scores with some criticisms in the biosecurity area, like the farms in the present study.

These results highlight the importance of context-specific evaluation frameworks that consider the unique characteristics of local breeds and small-scale, often marginal, farming systems. As such, welfare assessment tools must be sensitive enough to capture strengths and limitations specific to these contexts, without penalizing farms for structural, environmental, or technological factors inherent to traditional systems [39]. ClassyFarm can be a flexible welfare assessment as it can be applied to both LHS and THS; however, it may penalize small-scale farms of both HSs and located in different areas (e.g., mountainous vs. lowland regions) that lack the resources for investment, renewal, or technological innovation, often resulting in lower scores [40,41]. Moreover, small-scale farms often face logistical and economic constraints that may limit their ability to fully comply with standardized protocols, like ClassyFarm, especially in areas such as biosecurity, which may require infrastructural investments not always feasible in extensive or remote settings [6,8,28].

In this study, the ClassyFarm system only allowed the assessment of the Rendena breed scenario on a limited number of farms. The dataset was restricted both in terms of available information (e.g., pasture access) and the number of herds, which prevented a more detailed investigation of welfare scores and other factors related to milk performance and quality. In the future, a more balanced number of Rendena herds across HSs would improve the robustness of the analysis, although some imbalance is unavoidable in real-world data, especially for local breeds with restricted populations such as the Rendena.

Nonetheless, the ClassyFarm system is continuously evolving as scientific knowledge on animal welfare measurement advances, with indicators being updated or added to the checklists to enable more comprehensive evaluations in the field. In 2025, several pasture-related indicators were added to the management and structures areas to distinguish evaluations of outdoor vs. indoor environments. Future studies may benefit from these improvements to the ClassyFarm checklists, allowing for the detection of differences in welfare scores when comparing farming practices that include pasture with those that do not, as well as between periods when cows are kept outdoors vs. indoors within pasture-based systems, as suggested by Moriconi et al. (2024) [7].

## 4. Conclusions

In northeastern Italy, local Rendena cattle are managed either in LHS or in THS, the latter being typical of mountainous regions where winter housing involves tethering, while grazing is allowed during the non-winter period. Overall, a good level of welfare was found in the 17 Rendena herds included in the study. Despite the differences between LHS and THS, farmers in this context have achieved good welfare outcomes, likely reflecting the Rendena’s rusticity and adaptability, together with a high level of commitment and effective herd management. Significant differences were observed in milk yield, protein, and casein contents between the two HSs in Rendena cows, with LHS cows producing more milk and of higher quality in terms of these traits. These differences cannot be attributed solely to the HS, as other management- and nutrition-related factors may have contributed to the observed variation. Given that both milk yield and protein/casein content are key factors in cheesemaking, an essential objective for farmers raising the Rendena breed, these findings suggest that LHS conditions may confer a potential production advantage, within the limits of the information available in the present study. Further research is needed to disentangle the specific contribution of HS from other influencing factors.

## Figures and Tables

**Table 1:** Consistency of the 17 Rendena herds.

Consistency, *n*	Loose Housing System (*n* Herds = 5)		Tied Housing System (*n* Herds = 12)	
	Mean (SD)	Range	Mean (SD)	Range
Total cows	213.40 (67.77)	95–280	57.64 (24.33)	20–115
Lactating cows	101.70 (35.02)	33–125	30.45 (12.35)	10–56
Dry cows	20.08 (10.48)	2–27	6.86 (2.71)	4–13
Heifers	66.54 (26.85)	34–105	13.80 (7.42)	5–27
Calves	25.13 (4.43)	16–29	6.52 (6.72)	0–19

**Table 2:** Mean, SD, and range of the overall welfare and areas ^1^ scores (%) according to ClassyFarm evaluations within the type of housing system ^2^.

Welfare Score, %	Loose Housing System (*n* Herds = 5)		Tied Housing System (*n* Herds = 12)	
	Mean (SD)	Range	Mean (SD)	Range
Overall	79.14 (3.55)	76–87	80.60 (3.58)	69–85
Biosecurity area	49.94 (10.73)	37–85	51.15 (20.75)	23–87
Management area	76.13 (5.40)	48–96	79.94 (8.10)	66–95
Structures area	73.36 (4.92)	65–81	72.36 (9.83)	56–88
ABMs area	83.48 (7.22)	74–95	84.90 (4.92)	72–92
Major hazards	67.45 (11.84)	40–85	52.72 (21.77)	21–87

^1^ ABMs = animal-based measures. ^2^ No statistically significant differences were observed between the means from the *t*-tests.

**Table 3:** Descriptive statistics (mean, SD, and range) of milk traits ^1^ of Rendena cows included in this study.

Trait	Mean (SD)	Range
Milk yield, kg/d	19.58 (6.65)	1.00–43.30
Fat, %	3.62 (0.62)	1.50–6.64
Protein, %	3.37 (0.38)	2.38–4.80
Casein, %	2.66 (0.32)	1.77–3.85
Lactose, %	4.79 (0.21)	4.11–5.38
SCS	3.14 (1.77)	−3.64–9.59
DSCS	2.49 (2.03)	−2.96–9.34
Urea, mg/dL	22.20 (6.28)	3.00–43.10

^1^ SCS = somatic cell score; DSCS = differential somatic cell score.

**Table 4:** F values, significance ^1^, and least square means (standard error) of milk quality traits ^2^ for the fixed effect of housing system. Means with different superscript letters within the housing system are statistically significantly different (*p* ≤ 0.05).

Trait	F Values	Loose Housing System	Tied Housing System
Fat, %	0.04	3.71 ^a^ (0.06)	3.70 ^a^ (0.04)
Protein, %	6.87 **	3.50 ^a^ (0.03)	3.41 ^b^ (0.02)
Casein, %	7.04 **	2.77 ^a^ (0.02)	2.70 ^b^ (0.02)
Lactose, %	0.113	4.79 ^a^ (0.01)	4.79 ^a^ (0.01)
SCS	2.47	3.20 ^a^ (0.10)	3.01 ^a^ (0.08)
DSCS	3.58	2.62 ^a^ (0.10)	2.35 ^a^ (0.10)
Urea, mg/dL	4.21 *	21.70 ^b^ (1.10)	24.30 ^a^ (0.70)

^1^ ** *p* < 0.01; * *p* < 0.05. ^2^ SCS = somatic cell score; DSCS = differential somatic cell score.

## Data Availability

Dataset available on request from the authors.

## Supplementary Materials

The following supporting information can be downloaded at: https://www.mdpi.com/article/10.3390/ani16040636/s1, Table S1: Definition of the 105 ClassyFarm indicators of the loose housing system (LHS) checklist1. Indicators that are not present in the tied housing system (THS) checklist are marked by an asterisk (*); Table S2: Definition of the 99 ClassyFarm indicators of the tied housing system (THS) checklist1. Indicators that are not present in the loose housing system (LHS) checklist are marked by an asterisk (*).

## Abbreviations

The following abbreviations are used in this manuscript:

ABMs	Animal-based measures
AIA	Associazione Italiana Allevatori
ANARE	Associazione Nazionale Allevatori Bovini di Razza Rendena
ARAV	Associazione Regionale Allevatori del Veneto
CReNBA	National Reference Centre for Animal Welfare
DGSA	General Directorate of Animal Health and Veterinary Drugs
DSCC	Differential somatic cell count
DSCS	Differential somatic cell score
HS	Housing system
IZSLER	Istituto Zooprofilattico Sperimentale della Lombardia e dell’Emilia Romagna
LHS	Loose housing system
SCC	Somatic cell count
SCS	Somatic cell score
THS	Tied housing system
