# Supplementary material for: Evaluating Welfare, Milk Quality and Yield of Rendena Cows in Loose vs. Tied Housing Systems
Source: Animals (Basel). 2026 Feb 17;16(4):636. doi: 10.3390/ani16040636 (PMC12937469; doi:10.3390/ani16040636)
Supplement: Supplementary file 1 [file animals-16-00636-s001.zip › animals-4122600-supplementary.pdf]

Table S1. Definition of the 105 ClassyFarm indicators of the loose housing system (LHS) checklist[1]. Indicators not present in the tied housing system (THS) checklist are marked by an asterisk (*).

Area	Indicator n.	Indicator description
Biosecurity	1	Control of rodents and insects.
	2	Contact with other animal species.
	3	Measures to prevent the entrance of occasional visitors.
	4	Measures to control the entrance of regular visitors.
	5	Disinfection of vehicles (e.g. trucks, cars) upon entering the farm.
	6	Contact between external truck (e.g., feed truck) and cattle on farm.
	7	Carcass storage and removing.
	8	Loading of live animals (e.g., for sale).
	9	Animal purchasing or temporary exit of the animals from the farm (e.g., for exhibitions).
	10	Quarantine.
	11	Control and prevention of major infectious diseases.
	12	Health monitoring activities.
	13	Control and prevention of mammary infections.
	14	Control and prevention of endo/ectoparasitic infections.
	15	Analysis of drinking water.
Management	16	Number of stockpersons.
	17	Experience and training of stockpersons.
	18	Animal grouping strategy.
	19	Number of inspections (bovines > 6 months old).
	20	Number of inspections (calves).
	21	Treatment of sick or injured animals.
	22	On-farm culling procedures.
	23	Type of handling.
	24	Feeding strategy (bovines > 6 months old).
	25	Feed availability (bovines > 6 months old).
	26	Use of concentrate feeds (daily dose) (lactating cows).
	27	Colostrum feeding for calves.
	28	Management of feeds, daily ration, and feeding frequency (milk and fiber) (calves).
	29	Water availability (all groups).
	30	Cleanliness of drinkers (lactating cows).
	31	Cleanliness of drinkers (dry cows).
	32	Cleanliness of drinkers (heifers).
	33*	Cleanliness of floor in walking areas (lactating cows).
	34*	Cleanliness of floor in walking areas (dry cows).
	35*	Cleanliness of floor in walking areas (heifers).

	36	Cleanliness of bedding material and related management (lactating cows).
	37	Cleanliness of bedding material and related management (dry cows).
	38*	Cleanliness of bedding material and related management (calving pen).
	39*	Calving area management and use.
	40	Cleanliness of bedding material and related management (heifers).
	41	Cleanliness of bedding material and related management (calves).
	42	Foot inspection and foot bathing.
	43	Hygiene of milking parlor (or milking robot) and related equipment.
	44	Milking routine and udder hygiene.
	45	Biosecurity measures.
Structures	46	Absence of materials or equipment in the housing area (all groups).
	47	Presence of shelters for animals kept outside of the buildings (all groups).
	48	Type of housing for animals older than 6 months.
	49	Space availability in lying area (lactating cows).
	50	Space availability in lying area (dry cows).
	51*	Space availability in lying area (calving pen).
	52	Space availability in lying area (heifers).
	53*	Design of lying area (lactating cows).
	54	Type of bedding material (lactating cows).
	55	Type of bedding material (dry cows).
	56	Type of bedding material (heifers).
	57	Type of floor in walking areas (lactating cows).
	58	Type of floor in walking areas (dry cows).
	59	Type of floor in walking areas (heifers).
	60	Available space at feed bunk (lactating cows).
	61	Available space at feed bunk (dry cows).
	62	Available space at feed bunk (heifers).
	63*	Feeding place dimension and accessibility (lactating cows).
	64	Functioning and number of drinkers (lactating cows).
	65	Functioning and number of drinkers (dry cows).
	66	Functioning and number of drinkers (heifers).
	67	Bedding material for newborn calves in single pens.
	68	Space availability for calves in single pens.
	69	Possibility for calves in single pens to see and touch each other.
	70	Space availability for calves in group pens.
	71	Facilities for sick animals (all groups).
	72*	Milking parlor access and exit.
	73*	Waiting room and milking parlor design.

	74	Milking machine or robot maintenance.
	75	Temperature, humidity and ventilation.
	76	Gas (NH_3, H_2S, CO_2) concentration.
	77	Use of artificial lighting.
ABMs (animal-based measures)	78	Avoidance distance test (lactating cows).
	79	Avoidance distance test (dry cows).
	80	Avoidance distance test (heifers).
	81	Body condition score (lactating cows).
	82	Body condition score (dry cows).
	83	Body condition score (heifers).
	84	Cleanliness of flank, leg, udder (lactating cows).
	85	Cleanliness of flank, leg, udder (dry cows).
	86	Cleanliness of flank, leg, udder (heifers).
	87	Integument alterations (lactating cattle).
	88	Integument alterations (dry cows).
	89	Integument alterations (heifers).
	90	Lameness (adult cattle).
	91	Udder health- milk somatic cell count.
	92	Annual antibiotic treatment for clinical mastitis.
	93	Annual mortality rate (adult cattle).
	94	Annual mortality rate (heifers).
	95	Annual mortality rate (calves).
	96	Mutilations (all groups).
Major hazards	97	Drinking water sources.
	98	Noise level.
	99	Inspection lighting.
	100	Alarm system in case of mechanical ventilation disruption.
	101	Fire alarm.
	102	Inspection of automatic and mechanical equipment.
	103	Maintenance of the record of pharmacological treatments.
	104	Maintenance of the record for tracking animal movements.
	105	Illegal substances administration.

[1] (https://www.classyfarm.it/index.php/vet-aziendale-it , accessed 30 September 2024).

Table S2. Definition of the 99 ClassyFarm indicators of the tied housing system (THS) checklist[1]. Indicators not present in the loose housing system (LHS) checklist are marked by an asterisk (*).

Area	Indicator n.	Indicator description
Biosecurity	1	Control of rodents and insects.
	2	Contact with other animal species.
	3	Measures to prevent the entrance of occasional visitors.
	4	Measures to control the entrance of regular visitors.
	5	Disinfection of vehicles (e.g. trucks, cars) upon entering the farm.
	6	Contact between external truck (e.g., feed truck) and cattle on farm.
	7	Carcass storage and removing.
	8	Loading of live animals (e.g., for sale).
	9	Animal purchasing or temporary exit from the farm (e.g., for exhibitions).
	10	Quarantine.
	11	Control and prevention of major infectious diseases.
	12	Health monitoring activities.
	13	Control and prevention of mammary infections.
	14	Control and prevention of endo/ectoparasitic infections.
	15	Analysis of drinking water.
Management	16	Number of stockpersons.
	17	Experience and training of stockpersons.
	18	Animal grouping strategy.
	19	Number of inspections (bovines > 6 months old).
	20	Number of inspections (calves).
	21	Treatment of sick or injured animals.
	22	On-farm culling procedures.
	23	Type of handling.
	24	Feeding strategy (bovines > 6 months old).
	25	Feed availability (bovines > 6 months old).
	26	Use of concentrate feeds (daily dose) (lactating cows).
	27	Colostrum feeding for calves.
	28	Management of feeds, daily ration, and feeding frequency (milk and fiber) (calves).
	29	Water availability (all groups).
	30	Cleanliness of drinkers (lactating cows).
	31	Cleanliness of drinkers (dry cows).
	32	Cleanliness of drinkers (heifers).
	33	Cleanliness of bedding material and related management (lactating cows).
	34	Cleanliness of bedding material and related management (dry cows and cows at calving).

	35	Cleanliness of bedding material and related management (heifers).
	36	Cleanliness of bedding material and related management (calves).
	37	Foot inspection and foot bathing.
	38	Hygiene of milking equipment.
	39	Milking routine and udder hygiene.
	40	Biosecurity measures.
Structures	41	Absence of materials or equipment in the housing area (all groups).
	42	Presence of shelters for animals kept outside of the buildings (all groups).
	43	Type of housing for animals older than 6 months.
	44	Space availability in lying area (lactating cows).
	45	Space availability in lying area (dry cows and cows at calving).
	46	Space availability in lying area (heifers).
	47	Type of bedding material (lactating cows).
	48	Type of bedding material (dry cows).
	49	Type of bedding material (heifers).
	50	Type of floor in walking areas (lactating cows).
	51	Type of floor in walking areas (dry cows).
	52	Type of floor in walking areas (heifers).
	53*	Presence of electric trainers.
	54	Available space at feed bunk (lactating cows).
	55	Available space at feed bunk (dry cows).
	56	Available space at feed bunk (heifers).
	57	Functioning and number of drinkers (lactating cows).
	58	Functioning and number of drinkers (dry cows).
	59	Functioning and number of drinkers (heifers).
	60	Bedding material for newborn calves in single pens.
	61	Space availability for calves in single pens.
	62	Possibility for calves in single pens to see and touch each other.
	63	Space availability for calves in group pens.
	64	Facilities for sick animals (all groups).
	65	Milking equipment maintenance.
	66	Temperature, humidity and ventilation.
	67	Gas (NH_3, H_2S, CO_2) concentration.
	68	Use of artificial lighting.
ABMs (animal-based measures)	69	Avoidance distance test (lactating cows).
	70	Avoidance distance test (dry cows).
	71	Avoidance distance test (heifers).
	72	Body condition score (lactating cows).
	73	Body condition score (dry cows).
	74	Body condition score (heifers).
	75	Cleanliness of flank, leg, udder (lactating cows).
	76	Cleanliness of flank, leg, udder (dry cows).

	77	Cleanliness of flank, leg, udder (heifers).
	78	Integument alterations (lactating cows).
	79	Integument alterations (dry cows).
	80	Integument alterations (heifers).
	81	Lameness (adult cattle).
	82*	Prevalence of long and deformed hooves (adult cattle).
	83	Udder health- milk somatic cell count.
	84	Annual antibiotic treatment for clinical mastitis.
	85*	Lying behaviour (lactating cows).
	86*	Lying behaviour (dry cows).
	87	Annual mortality rate (adult cattle).
	88	Annual mortality rate (heifers).
	89	Annual mortality rate (calves).
	90	Mutilations (all groups).
Major hazards	91	Drinking water sources.
	92	Noise level.
	93	Inspection lighting.
	94	Alarm system in case of mechanical ventilation disruption.
	95	Fire alarm.
	96	Inspection of automatic and mechanical equipment.
	97	Maintenance of the record of pharmacological treatments.
	98	Maintenance of the record for tracking animal movements.
	99	Illegal substances administration.

[1] (https://www.classyfarm.it/index.php/vet-aziendale-it , accessed 30 September 2024).
